# 2-Methylquinazoline derivative 23BB as a highly selective histone deacetylase 6 inhibitor alleviated cisplatin-induced acute kidney injury

**DOI:** 10.1042/BSR20191538

**Published:** 2020-01-17

**Authors:** Yan Hao, Fan Guo, Zhuo Huang, Yuying Feng, Zijing Xia, Jing Liu, Lingzhi Li, Rongshuang Huang, Lin Lin, Liang Ma, Ping Fu

**Affiliations:** 1 Division of Nephrology and National Clinical Research Center for Geriatrics, Kidney Research Institute, West China Hospital of Sichuan University, Chengdu 610041, China; 2 Division of Nephrology, No. 1 People Hospital of Zigong, Zigong 643000, China; 3 Division of West-district Outpatient, West China Hospital of Stomatology, Sichuan University, Chengdu 610041, China

**Keywords:** Acute kidney injury, Apoptosis, Cisplatin, Endoplasmic reticulum stress, Histone deacetylase 6 inhibitor

## Abstract

Histone deacetylases 6 (HDAC6) has been reported to be involved in the pathogenesis of cisplatin-induced acute kidney injury (AKI). Selective inhibition of HDAC6 might be a potential treatment for AKI. In our previous study, a highly selective HDAC6 inhibitor (HDAC6i) 23BB effectively protected against rhabdomyolysis-induced AKI with good safety. However, whether 23BB possessed favorable renoprotection against cisplatin-induced AKI and the involved mechanisms remained unknown. In the study, cisplatin-injected mice developed severe AKI symptom as indicated by acute kidney dysfunction and pathological changes, companied by the overexpression of HDAC6 in tubular epithelial cells. Pharmacological inhibition of HDAC6 by the treatment of 23BB significantly attenuated sCr, BUN and renal tubular damage. Mechanistically, 23BB enhanced the acetylation of histone H3 to reduce the HDAC6 activity. Cisplatin-induced AKI triggered multiple signal mediators of endoplasmic reticulum (ER) stress including PERK, ATF6 and IRE1 pathway, as well as CHOP, GRP78, p-JNK and caspase 12 proteins. Oral administration of our HDAC6i 23BB at a dose of 40 mg/kg/d for 3 days notably improved above-mentioned responses in the injured kidney tissues. HDAC6 inhibition also reduced the number of TUNEL-positive tubular cells and regulated apoptosis-related protein expression. Overall, these data highlighted that HDAC6 inhibitor 23BB modulated apoptosis via the inhibition of ER stress in the tubular epithelial cells of cisplatin-induced AKI.

## Introduction

Acute kidney injury (AKI), characterized by a rapid decline of glomerular filtration rate, is a clinical syndrome correlated with a high mortality and the increased risk of chronic kidney diseases (CKD) [1,2]. Cisplatin, as a commonly used tumor chemotherapeutic drug, possessed a dose-limited side effect of nephrotoxicity [3]. Cisplatin must be withheld from patients because of confounding factors predisposing them to an increased risk for kidney injury, including smoking, hypoalbuminemia, advanced age or known CKD. Approximately 30% of cisplatin-administered patients suffered from renal dysfunction and injury, especially AKI. Unfortunately, treatment strategy for cisplatin-induced kidney diseases remain limited [4].

Although the involved mechanisms have not been fully explored, the endoplasmic reticulum (ER) stress-mediated apoptosis played crucial roles in cisplatin-induced AKI [5–8]. Recently, ER stress emerged as a key pathophysiological paradigm underlying apoptosis. The presence of misfolded proteins and stresses led to the activation of an adaptive program in the ER, which also known as unfolded protein response (UPR), to restore protein-folding homeostasis. Initiation of canonical UPR engaged three key signaling pathways, which was mediated by inositol-requiring enzyme-1 (IRE1), double-stranded RNA-activated protein kinase-like ER kinase (PERK) and activating transcription factor-6 (ATF6). The UPR was also linked to the activation of stress kinases such as the c-Jun N-terminal kinase (JNK) and splicing of X-box binding protein 1 (XBP1) [5]. In the physiological conditions, signal proteins of ER stress (PERK, IRE1, ATF6) were bound by ER chaperone GRP78 and maintained inactive state. Under ER stress, these ER proteins were dissociated from GRP78 and then activated signal transduction to inhibit protein translation, up-regulate molecular chaperones to enhance protein-folding capacity, and/or promote unfolded or misfolded proteins degradation by ER-related protein degradation (ERAD) pathway [9]. Functionally, depending on the severity and duration of ER stress, UPR could be adaptive or trigger cell death. When cells under excessive or prolonged ER stress failed to resolve the protein-folding defect and restore homeostasis in the ER via adaptive UPR pathway, apoptosis was triggered [10–13].

Multiple cellular functions, including cell apoptosis, were modulated by the acetylation of histone and non-histone proteins. An acetylation could be added to a lysine residue by histone acetyl transferases and be cleaved by histone deacetylases (HDACs) [14]. HDACs were classified based on their structure and homology: class I (HDAC1, 2, 3 and 8); class II (HDAC4, 5, 6, 7, 9 and 10); class III (SIRT1-7); and class IV (HDAC11) [15]. Among these isoforms of HDACs, HDAC6 has been confirmed to contribute to the pathogenesis of rhabdomyolysis and cisplatin-induced AKI by the activation of tubular cell apoptosis, inflammatory response, macrophage infiltration and oxidative stress in kidneys [16,17]. Therefore, selective inhibition of renal HDAC6 activity may be a promising option for AKI treatment.

In our previous study, N-hydroxy-4-(2-methoxy-5-(methyl(2-methylquinazolin-4-yl)amino)phenoxy)butanamide (23BB, Supplementary Figure S1) as a highly selective HDAC6 inhibitor has been designed, synthesized and substantiated by anti-tumor activity in both solid and hematologic tumor models with good safety [18]. Our previous data also highlighted that HDAC6I 23BB significantly alleviated rhabdomyolysis-induced AKI that was substantiated by the reduction of ER stress-mediated apoptosis in tubular epithelial cells [19,20]. However, the potential of HDAC6i 23BB as a potential drug candidate in cisplatin-induced AKI had not been investigated. In the study, we aimed to explore whether 23BB improved cisplatin-induced AKI by inhibiting HDAC6 activity and to determine the involved mechanisms.

## Materials and methods

### Animals

The study adhered to the “Principles of Laboratory Animal Care” (National Institutes of Health publication 85-23, revised 1985) that minimized both the number of animals used and any suffering that they might experience, and was approved by Animal Care and Use Ethics Committee of Sichuan University (IACUC number: 2017080A). The 8-week old male C57BL/6J mice were purchased from the Animal Laboratory Center of Sichuan University (Chengdu, China) and housed in a controlled environment (constant temperature at 20 ± 2°C and humidity at 50–60% with a 12-h light and 12-h dark cycle) and were free access to standard laboratory food and water. The mice were adapted for 1 week before further research. The animal experiments were taken place in the Animal Experiment Center of West China Hospital.

### Primary antibodies

Anti-HDAC6 (sc11420, Santa Cruz), anti-acetyl-histone H3 (9649, Cell Signaling Technology), Anti-H3 (ab8580, Abcam), anti-BAX (ab32503, Abcam), anti-BCL-2 (ab3214, Abcam), anti-BCL-XL (ab32370, Abcam), anti-cleaved caspase 3 (66470-2-Ig, Proteintech Group), anti-phospho-JNK (4668, Cell Signaling Technology), anti-JNK (ab208035, Abcam), anti-phospho-eIF2α (ET1603-14, HuaBio), anti-eIF2α (RT1196, HuaBio), anti-phospho-PERK (sc32577, Santa Cruz), anti-GRP78 (ER40402, HuaBio), anti-IRE1α (ab48187, Abcam), anti-CHOP (2895, Cell Signaling Technology), anti-ATF4 (11815, Cell Signaling Technology), Anti-ATF6 (ER1706-34, HuaBio), anti-XBP1 (ab37152, Abcam), anti-caspase 12 (ab62484, Abcam). Primer sequences was exhibited in Supplementary Material (Supplementary Table S1).

### Cisplatin treatment of mice

Mice were intraperitoneally injected with 20 mg/kg body weight cisplatin [21], and control mice received an intraperitoneal injection of equal volume of 0.9% saline [22]. As for the HDAC6i 23BB group, HDAC6i was dissolved in PEG_400_, diluted in 0.9% saline and orally administered at a dose of 40 mg/kg/d for 3 days before the cisplatin injection. Mice were killed via pentobarbital sodium injection (50 mg/kg, i.p.) after 3 days, blood sample was collected for serum creatinine (sCr) and blood urea nitrogen (BUN) measurement, the upper half of the left kidney was removed and fixed in 10% phosphate-buffered formalin for PAS staining and TUNEL assay. The lower half of the left kidney was fixed in 2.5% glutaraldehyde for 2 h at 4°C and processed for transmission electron microscope. The right kidney was removed and frozen in liquid nitrogen, then stored at −80°C.

### Serum analysis

The sCr and BUN were examined by automatic biochemical analyzer (Mindray BS-240, Shenzhen, China). The AKI mice model was considered to be established when the level of sCr of the cisplatin-treated group rose up to two times of their control littermates.

### Histologic examination

The kidney, fixed in 10% phosphate buffered formalin, was dehydrated in a graded series of alcohol concentrations and embedded in paraffin. Kidney blocks were cut into 4 μm sections and then subjected to PAS staining for morphologic analysis. PAS-stained tissue was viewed by light microscopy at magnifications of ×200 or ×400. For semiquantitative analysis of morphological changes, two sections were randomly selected from each sample of at least 3 for every group and 10 fields were randomly selected at a magnification of ×200 from each section in PAS staining. Histopathological changes were evaluated by the percentage of injured/damaged renal tubules, as indicated by tubular lysis, dilation, disruption and cast formation. Tissue injury was scored on a scale of 0-4, with 0, 1, 2, 3 and 4 corresponding to 0%, <25%, 26–50%, 51–75% and >76% of injured/damaged renal tubules, respectively. Ten field of ×40 magnification was examined and averaged.

### Immunoblot analysis

Proteins were extracted from kidney tissues or HK-2 cells using RIPA lysis buffer containing 4% cocktail proteinase inhibitors (P0013B, Beyotime Biotechnology, China). After centrifugation at 13,000 rpm for 15 min at 4°C, the supernatant was collected, and protein concentrations were determined using the Pierce™ BCA Protein Assay Kit (23225, Thermo Scientific, Billerica, MA, United States). Bovine serum albumin was used as the standard. Equal amounts of protein lysate were loaded directly on 10–12% SDS-PAGE, transferred onto polyvinylidene difluoride (PVDF) Membrane for Protein Blotting (162-0177, Bio-Rad, Hercules, CA, United States). The membranes were blocked with 5% non-fat dry milk (w/v) in Tris-buffered saline with 0.1% Tween-20 (TBS-T) for 1 h at room temperature and then incubated with indicated primary antibodies overnight at 4°C. After being rinsed thrice with TBS-T at 5-min intervals, the membranes were incubated with horseradish peroxidase-labeled goat anti-rabbit IgG (HA1001, 1:2000 dilution; HuaBio, Hangzhou, China) or goat anti-mouse IgG (HA1006, 1:2000 dilution; HuaBio, Hangzhou, China) for 1 h. Immunoblots were visualized using the Immobilon Western Chemiluminescent HRP Substrate (WBKLS0500, Millipore Corporation, Billerica, MA, United States) with Bio-Rad Chemi Doc MP. All immunoblot analysis data are performed in triplicate. Densitometry analysis was performed using ImageJ 6.0 software (National Institutes of Health, Bethesda, MD, United States).

### Immunofluorescence staining

Renal specimens were embedded in optimum cutting temperature (OCT) compound, frozen in acetone dry ice mixture and cut into 3–5 μm sections on a cryostat and stored at −80°C until use. Non-specific binding sites were blocked with phosphate-buffered saline (PBS) containing 5% bovine serum for 1 h at room temperature. For staining, we incubated the specimens overnight with first primary antibody at 4°C. After washing with PBS, the corresponding secondary antibody was applied for 1 h. After washing with PBS, the corresponding secondary antibody was applied for 1 h. The samples were washed with PBS, stained with DAPI (D8200, Solarbio, Beijing, China) and mounted with cover slips. In negative controls, primary antibodies were replaced by PBS. Secondary antibodies (1:500 dilution; Jackson ImmunoResearch) matched with a corresponding primary antibody were used to display fluorescent signals. Images were exported from ZEN 2012 (blue edition) microscopy software.

### Electron microscopy

After being fixed in cold 2.5% glutaraldehyde for 2 h at 4°C, kidney tissues were washed with phosphate-buffered saline (PBS) (0.2 mol/l, pH 7.4) for 2 h, fixed with 1% osmic acid for 2 h, and then washed six times with PBS for 10 min per wash. The samples were dehydrated with ethanol and cleaned with epoxypropane. They were embedded in EPON 812 overnight at room temperature. Ultrathin sections (40–60 nm) were cut (EM UC61rt, Leica) and stained with uranyl acetate/lead citrate. These sections were subsequently visualized using a transmission electron microscope (H-7650, Hitachi).

### Quantitative real-time PCR analysis

Total RNA from kidney tissues was extracted using a total RNA extraction Kit (TP-01121, Foregene, Chengdu, China) according to the protocols. The concentration of mRNA was tested using a Scan Drop 100 (Analytik Jena, Thuringia, Germany) determiner. Quantitative real-time PCR was performed after reverse transcription by using the fast qPCR kit (KK4610, Kapa Biosystems, Foster, CA, United States) in a PCR system (CFX Connect; Bio-Rad, Hercules, CA, United States). Relative expression levels were normalized to GAPDH.

### TUNEL assay

*In vivo*, the terminal deoxynucleotidyl transferase-mediated dUTP nick end labeling (TUNEL) staining was conducted on paraffin-embedded slides using the DeadEnd™ Fluorometric TUNEL System (G3250, Promega, Madison, Wisconsin, United States) according to the experimental protocol. The sections were then incubated with DAPI (D8200, Solarbio, Beijing, China) at a dilution of 1:500. Images were exported by fluorescence microscopy at magnifications of ×400. Positive cells were counted, and at least 10 fields per section for each sample were examined. *In vitro*, TUNEL assay was performed using the One Step TUNEL Apoptosis Assay Kit (C1086, Beyotime Biotechnology, China) according to the experimental protocol.

### Cell culture and cisplatin treatment

Human renal proximal tubule cell line (HK-2 cell) was a gift from Prof. Xueqing, Yu (The first Affiliated Hospital, Sun Yat-sen University) and maintained in Dulbecco’s modified Eagle’s medium (DMEM)/F12 (SH30023.01B, Hyclone, Beijing, China) supplemented with 10% fetal bovine serum (FBS) (SH30084.03, Hyclone, Australia) at 37°C under humidified atmosphere of 5% CO_2_ and 95% air. We divided the cells in exponential growth state into five groups: control, cisplatin (20 μg/ml); cisplatin + HDAC6i group, incubated with 23BB at 20 nM 30 min prior to cisplatin treatment; cisplatin + sodium 4-phenylbutyrate (4-PBA) (11323, Cayman Chemical, Ann Arbor, Michigan, United States), incubated with 4-PBA at 2 mM 30 min prior to cisplatin treatment; cisplatin + tunicamycin (11445, Cayman Chemical, Ann Arbor, Michigan, United States), incubated with tunicamycin (TM) at 25 ng/ml 30 min prior to cisplatin treatment; and control group, cells were incubated with complete medium alone in the control group.

### Annexin V-FITC/propidium iodide assay

An Annexin V- FITC apoptosis analysis kit (AO2001-02P-H, SUNGENE, Tianjin, China) was used. HK-2 cells were seeded in six-well plates. The adhered cells were exposed to different media for 24 h and then detached with trypsin. Subsequently, the cells were resuspended in a binding buffer and stained with Annexin V-FITC (5 μL) and propidium iodide (PI; 5μL) at room temperature for 15 min in the dark. Apoptotic cells were determined using a flow cytometer (Beckman Cytoflex, Beckman Coulter Australia Pty Ltd., Lane Cove, NSW, Australia). Annexin V-FITC-positive and PI-negative cells were considered apoptosis.

### Statistical analysis

All data are presented as the mean ± SEM. Data were analyzed by one-way ANOVA using SPSS 17.0 (SPSS Institute Inc., Chicago, IL, United States). Comparisons between two groups were evaluated by an unpaired Student’s *t*-test. The *P* value < 0.05 was considered statistically significant.

## Results

### HDAC6i 23BB protected against cisplatin-induced AKI

**Figure 1:**
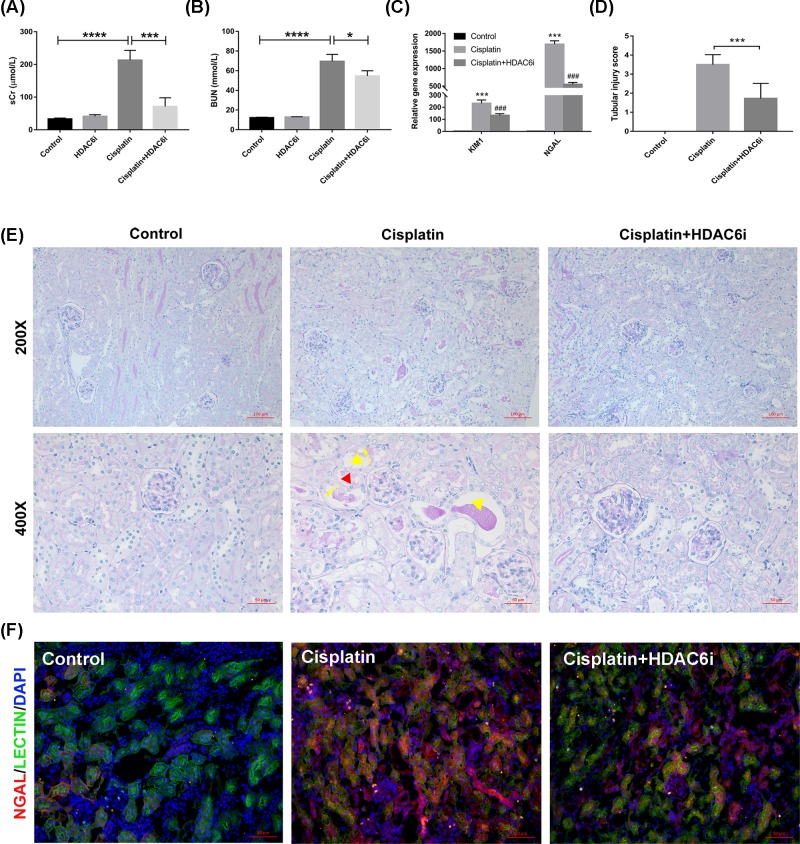
Treatment by HDAC6 inhibitor alleviated cisplatin-induced AKI (**A** and **B**) serum creatinine (sCr) and blood urea nitrogen (BUN). (**C**) Relative mRNA expression of KIM1 and NGAL in kidney tissues. (**D**) Tubular injury score and (**E**) periodic acid-Schiff (PAS) staining of the kidney tissues (200× and 400×). Red triangle: tubular dilatation; yellow triangle: cast formation; yellow arrow: loss of brush border. (**F**) Immunofluorescence staining of NGAL and Lectin in the kidney tissue. NGAL was used as an AKI marker, and Lectin as a marker of tubular epithelial cells. All data are represented as the means ± SE (*n* = 6); *P < 0.05, ***P < 0.001, ****P < 0.0001 vs. Control, ^###^*P* < 0.001 vs. Cisplatin.

To confirm whether HDAC6i 23BB possess renoprotective effect, we evaluated renal function and pathological changes of kidney tissues in a mouse model of cisplatin-induced AKI. As exhibited in Figure 1, serum creatinine (sCr), blood urea nitrogen (BUN), renal mRNA levels of KIM1 and NGAL were markedly elevated at 3 days after cisplatin injection. Treatment of HDAC6i at a dose of 40 mg/kg/d for 3 days significantly improved acute renal dysfunction with good safety (Supplementary Figure S2). Consistently, the result of PAS-stained kidneys showed less tubular dilatation, swelling, necrosis, cast formation and preservation of a brush border in the HDAC6i-treated mice as compared with that of cisplatin-induced group (Figure 1D,E). Immunofluorescence staining of renal injury maker NGAL in the tubular epithelial cells (Lectin) of kidney tissues further confirmed that oral administration of HDAC6i alleviated cisplatin-induced AKI (Figure 1F).

### Inhibition of tubular HDAC6 activity by 23BB enhanced the acetylation of histone H3 and α-tubulin in kidney of cisplatin-induced AKI

**Figure 2:**
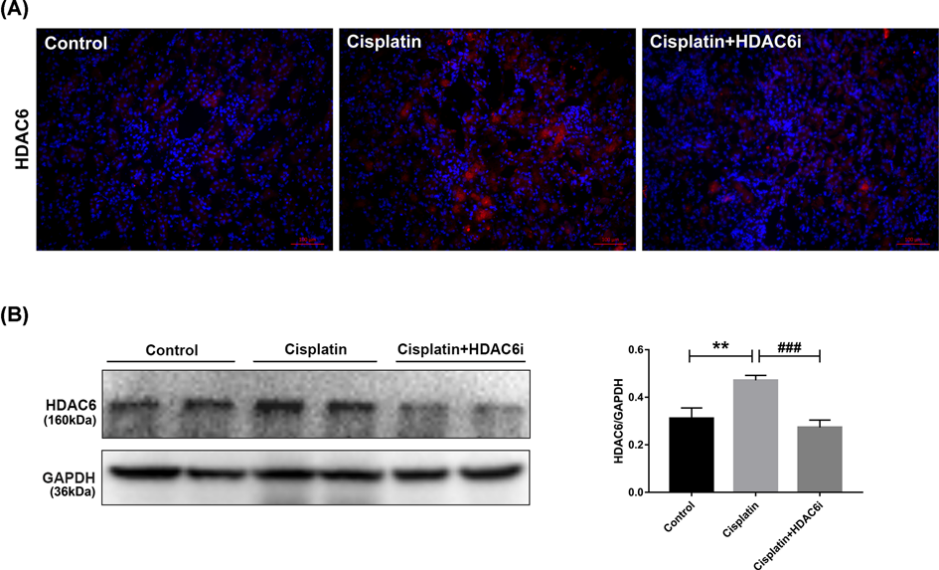
The expression of HDAC6 in the kidney tissue of cisplatin-induced AKI (**A**) Immunofluorescence staining of HDAC6 in the kidney tissue. (**B**) The kidney tissue lysates were subjected to immunoblot analysis with indicated antibodies against HDAC6. Expression of HDAC6 was quantified by densitometry and normalized with GAPDH. Data are represented as the means ± SE (*n* = 3). **P < 0.01 vs. Control, ^###^*P* < 0.001 vs. Cisplatin.

To determine whether 23BB exhibited renoprotective effect by targeting HDAC6, we further evaluated the HDAC6 activity in kidney tissues of cisplatin-induced AKI. As shown in Figure 2, renal HDAC6 expression was markedly elevated at 3 days after cisplatin injection, and treatment of HDAC6i at a dose of 40 mg/kg/d for 3 days significantly inhibited HDAC6 protein expression in the injured kidney tissue by immunofluorescence staining and Western blotting.

**Figure 3:**
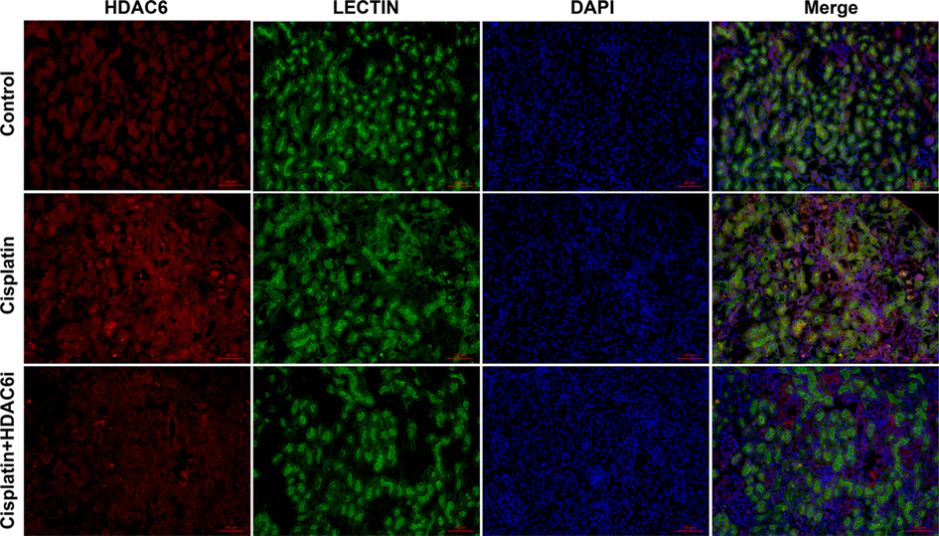
Immunofluorescence staining of HDAC6 in the tubular epithelial cells of cisplatin-induced AKI Lectin was used as a marker of tubular epithelial cells (HDAC6, red; Lectin, green; DAPI, blue).

Increasing evidence showed that tubular epithelial cells played diverse roles in renal repair or progression to AKI and chronic kidney disease (CKD). To further investigate whether HDAC6 was expressed in renal tubular epithelial cells, renal tissues were stained by HDAC6 and a proximal epithelial cell marker Lectin. As exhibited in Figure 3, HDAC6 was rarely expressed in the control group, but notably overexpressed in cisplatin-injected group and mainly merged with Lectin. Oral administration of HDAC6i significantly inhibited the HDAC6 expression, which was consistent with the immunoblot analysis results. So, these findings suggested that cisplatin induced the HDAC6 overexpression in tubular epithelial cells, and 23BB suppressed the HDAC6 activity.

**Figure 4:**
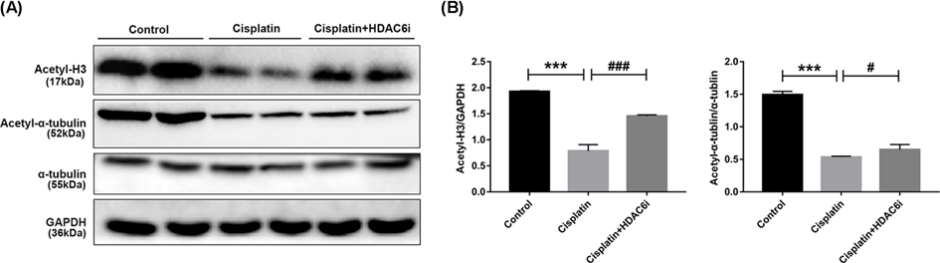
Inhibition of HDAC6 enhanced the acetylation of histone H3 and α-tubulin (**A**) The kidney tissue lysates were subjected to immunoblot analysis with indicated antibodies. (**B**) Expressions of acetylated histone H3 and α-tubulin were quantified by densitometry and normalized with β-actin. Data are represented as the means ± SE (*n* = 3). ***P < 0.001 vs. Control; **^#^***P* < 0.05, **^###^***P* < 0.001 vs. Cisplatin.

Inhibition of histone deacetylase was reflected by the increased expression of acetyl histone H3 and α-tubulin. To understand the inhibitory effect of 23BB, we investigated the expression of acetyl histone H3 and acetyl α-tubulin by immunoblot analysis. As exhibited in Figure 4, the reduced expressions of acetyl histone H3 and acetyl α-tubulin were detected in cisplatin-injected group, which were increased by HDAC6i treatment, especially acetyl histone H3 up-regulation.

### Inhibition of HDAC6 suppressed renal endoplasmic reticulum stress in cisplatin-induced AKI

**Figure 5:**
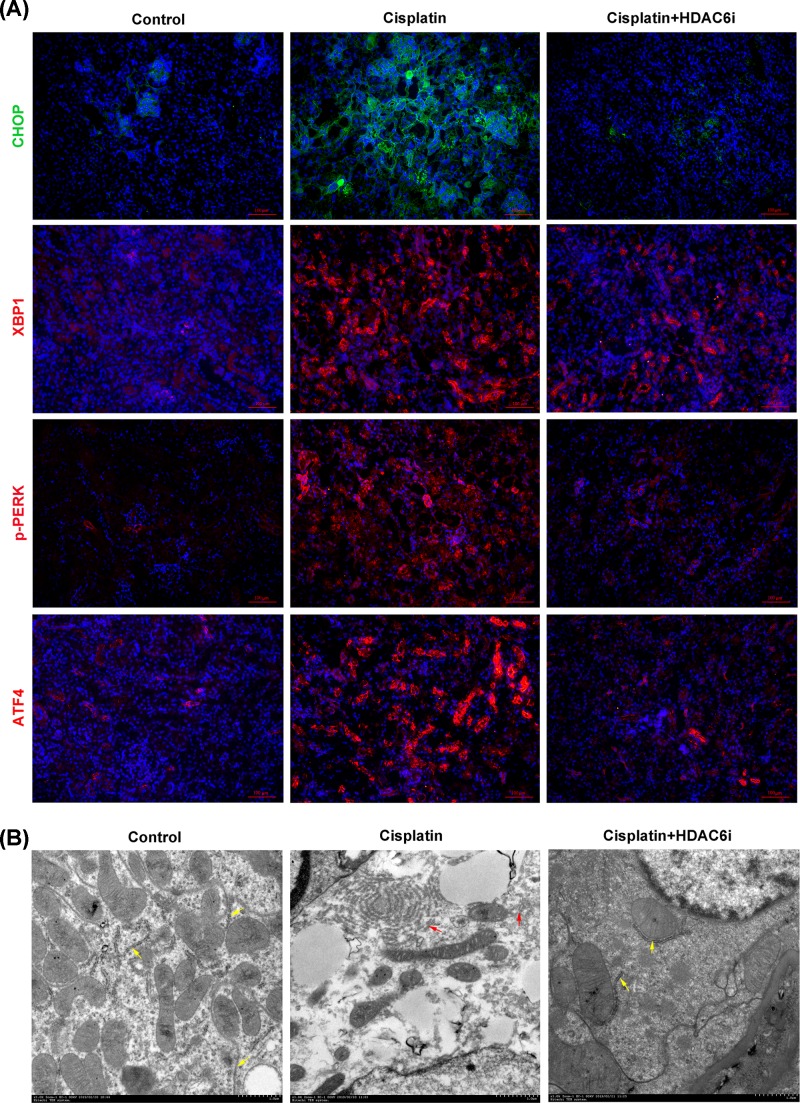
Inhibition of HDAC6 attenuated ER stress (**A**) Immunofluorescence staining of ER stress related CHOP, XBP1, p-PERK and ATF4 treated by HDAC6i in cisplatin-induced AKI. (**B**) Photomicrographs (30K) were collected by transmission electron microscope. Yellow arrow: normal endoplasmic reticulum; red arrow: expansion of endoplasmic reticulum.

Perturbations of kidney cells in AKI resulted in the accumulation of unfolded and misfolded proteins in the ER, leading to UPR or ER stress. First, we evaluated the levels of ER stress in kidney tissues by examining the expression of four UPR-related proteins, CHOP, XBP1, p-PERK and ATF4, which was found that these proteins were highly expressed in cisplatin-injured kidney tissues. After HDAC6i treatment, the expression of four proteins was effectively suppressed by immunofluorescence staining (Figure 5A). By transmission electron microscopy (Figure 5B), in the cisplatin group, we observed large amount of swelling ER in the cytoplasm of renal tubular cells, which was not detected in the control group. The administration of 23BB dramatically reduced swelling ER, while the mitochondrial injury and the brush border loss were still detected.

**Figure 6:**
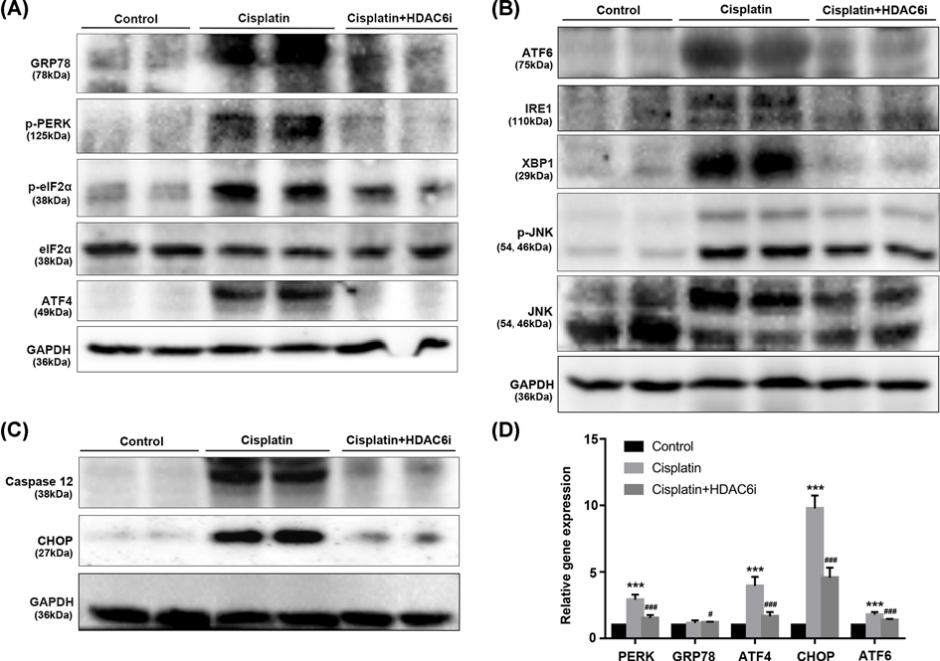
Inhibition of HDAC6 activity down-regulated renal ER stress related protein and mRNA expression (**A**) The expression of GRP78, p-PERK, p-eIF2α, eIF2α and ATF4 as measured by immunoblot analysis, in kidney tissue sections in each group. (**B**) The expression of ATF6, IRE1, XBP1 and p-JNK/JNK as measured by immunoblot analysis, in kidney tissue sections in each group. (**C**) The expression of caspase-12 and CHOP as measured by immunoblot analysis, in kidney tissue sections in each group. (**D**) Relative mRNA expression of PERK, GRP78, ATF4, CHOP and ATF6 in cisplatin-induced kidney tissues. All data are represented as the means ± SE (*n* = 6). ***P < 0.001 vs. Control; **^#^***P* < 0.05, **^###^***P* < 0.001 vs. Cisplatin.

To validate the hypothesis that HDAC6i alleviated cisplatin-induced AKI through the suppression of ER stress, we conducted immunoblot analysis of ER stress-related three pathways of IRE1, PERK and ATF6, as well as JNK, caspase 12. As shown in Figure 6A, the phosphorylation PERK and eIF2α was induced and the downstream ATF4 was also highly expressed in the cisplatin-injured kidney tissues. After HDAC6i treatment, the key protein expression of PERK pathway was effectively suppressed (Supplementary Figure S3). In Figure 6B, we also found that the expression of IRE1-XBP1 and ATF6 was markedly elevated in kidney at 3 days after cisplatin injection. Treatment of HDAC6i at a dose of 40 mg/kg/d for 3 days significantly improved ER tress. In addition, HDAC6i reduced cisplatin-induced ER-stress related mRNA level of PERK, ATF4 and ATF6 (Figure 6D and Supplementary Figure S3). Taken together, these findings suggested that cisplatin induced renal ER stress and HDAC6i 23BB administration inhibited renal ER stress activation in cisplatin-induced AKI.

**Figure 7:**
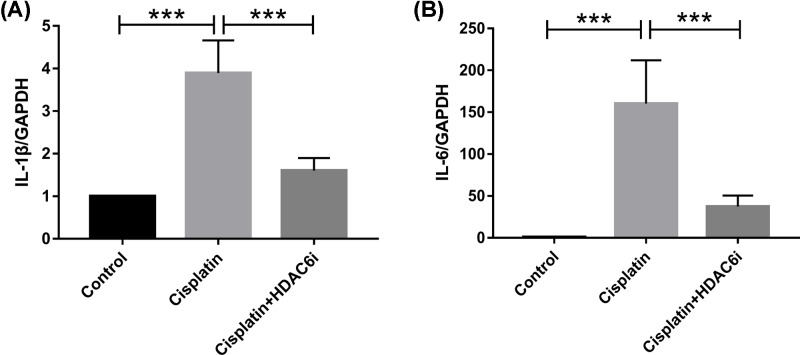
Inhibition of HDAC6 suppressed renal inflammatory cytokine mRNA expression of IL-1β and IL-6 (**A**) The mRNA values of IL-1β in the kidneys were normalized by the levels of GAPDH. (**B**) The mRNA values of IL-6 in the kidneys were normalized by the levels of GAPDH. Data are represented as the means ± SE (*n* = 6); ***P < 0.001.

The IRE1 complex activated the c-Jun N-terminal kinase (JNK) pathway to play a role in inflammation. The JNK phosphorylation (Figure 6B) with proinflammatory cytokine mRNA level of IL-1β and IL-6 (Figure 7) was also induced in the cisplatin-injured kidneys. Meanwhile, HDAC6i reduced the cisplatin-induced p-JNK protein expression and alleviated renal inflammation.

Several components of caspase cascade were involved in ER stress-induced apoptosis. Particularly, caspase 12 that was related with the ER membrane was a proximal regulator for ER stress-induced caspase activation followed by apoptosis. As shown in Figure 6C, cisplatin injection significantly induced the expression of caspase 12 and HDAC6i treatment effectively inhibited its expression in kidney (Supplementary Figure S3).

### Inhibition of HDAC6 reduced apoptosis in the kidneys of cisplatin-injured AKI

**Figure 8:**
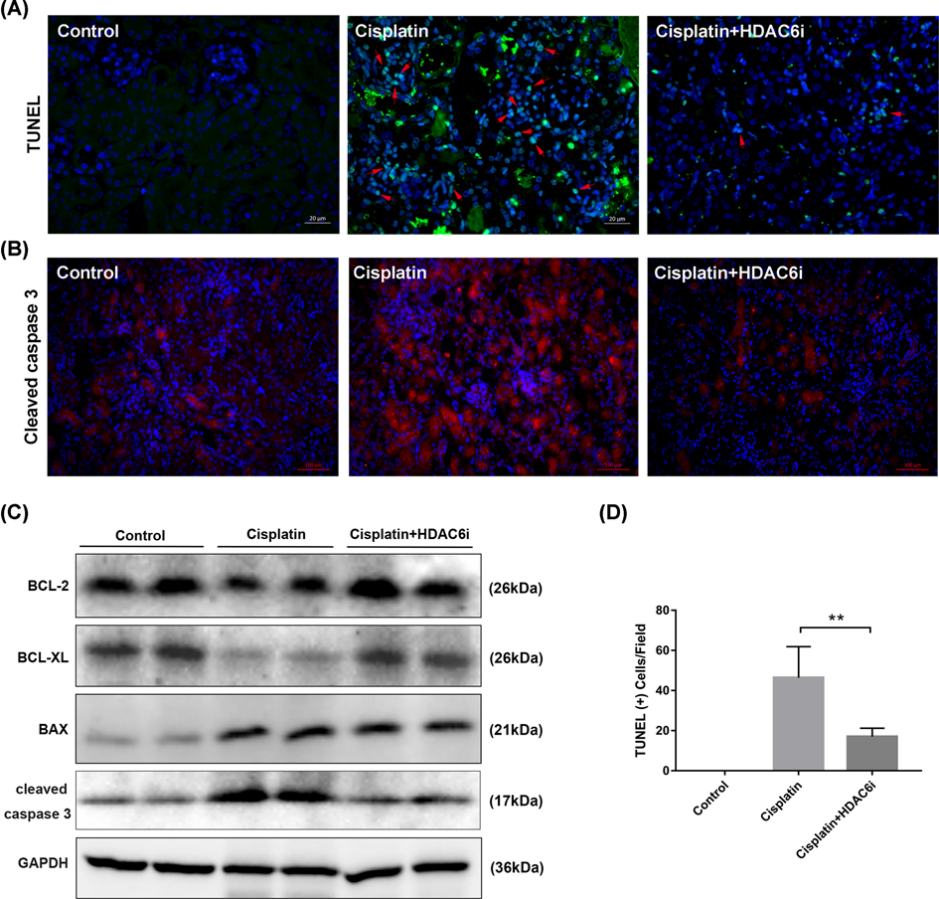
Inhibition of HDAC6 activity attenuated cisplatin-induced apoptosis in the kidney of AKI (**A**) TUNEL staining of kidney tissues (green: TUNEL positive cells, red arrow, ×400). (**B**) Immunofluorescence staining was performed to detect the expression of cleaved caspase 3 in kidney tissue sections (red, ×400). (**C**) The kidney tissue lysates were subjected to immunoblot analysis with indicated antibodies against BCL-2, BCL-XL, BAX and cleaved caspase 3. (**D**) The analysis of TUNEL positive cells.

Renal tubular cell apoptosis was a prominent feature in cisplatin-induced AKI. To investigate the role of HDAC6i in the model, we analyzed the cell apoptosis by TUNEL staining. As expected in Figure 8A, a number of TUNEL-positive cells were observed in the cisplatin-injured kidney and 23BB administration diminished the number of TUNEL-positive cells compared with that of cisplatin group.

ER stress could activate the caspase-12-dependent and the JNK-mediated apoptotic pathway. Caspase 3 was the key executioner that modified proteins responsible for apoptosis. Activated caspase 12 cleaves procaspase 9 to activate procaspase-3. In Figure 8B, the results of immunofluorescence staining showed that cisplatin induced the high expression of cleaved caspase 3 in kidney tissues and HDAC6i treatment significantly reduced its level.

We furtherly investigated the protein expression of BCL-2, BCL-XL, BAX and cleaved caspase 3 in the cisplatin-injured kidneys with or without 23BB treatment by Western blotting (Figure 8C and mRNA in Supplementary Figure S4). Consistent with our above-mentioned results, injection of cisplatin induced cell apoptosis, as assessed by the up-regulation of BAX and cleaved caspase 3, and the down-regulation of BCL-2 and BCL-XL. However, HDAC6i administration provided a marked renal protection against cisplatin-induced apoptosis, by restoring the expression of apoptosis-related signal markers.

### 23BB inhibited HDAC6 activity to improve ER stress-induced apoptosis in cisplatin-stimulated HK-2 cells

**Figure 9:**
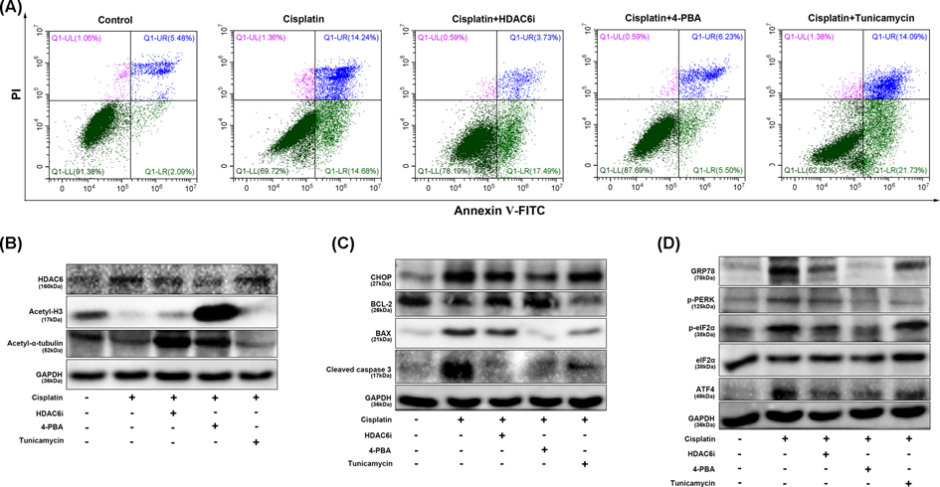
Inhibition of HDAC6 activity suppressed apoptosis and ER stress in cisplatin-induced renal proximal tubule HK-2 cells (**A**) HK-2 cells were treated with 23BB, subjected to Annexin-V/PI staining, and then analyzed the apoptosis by flow cytometry. (**B**) The expression of HDAC6, acetylated histone H3 and acetylated α-tubulin in HK-2 cells as measured by immunoblot analysis. (**C**) The expression of CHOP, BCL-2, BAX and cleaved caspase 3 in HK-2 cells as measured by immunoblot analysis. (**D**) The expression of GRP78, p-PERK, p-eIF2α/eIF2α and ATF4 in HK-2 cells as measured by immunoblot analysis.

Furthermore, we explored whether 23BB *in vitro* suppressed ER stress and apoptosis in cisplatin-stimulated human proximal tubule epithelial HK-2 cells. 4-PBA is a chemical chaperon to attenuate ER stress, and Tunicamycin is a naturally occurring antibiotic to induce ER stress in cells. After cisplatin stimulation, HK-2 cells showed a significant cell apoptosis and HDAC6i 23BB inhibited the number of apoptotic cells (Figure 9A and Supplementary Figure S5). Meanwhile, ER stress inhibitor 4-PBA also reduced the number of apoptotic cells and ER stress inducer Tunicamycin aggravated the apoptosis as compared with that of cisplatin.

As shown in Figure 9B, the results indicated that 23BB *in vitro* suppressed the HDAC6 expression by the regulation of acetyl α-tubulin. In the cisplatin-stimulated HK-2 cells, the protein expressions of two UPR molecule CHOP, GRP78 and the ER PERK-eIF2-ATF4 pathway as well as apoptosis-related cleaved caspase 3 were induced. HDAC6i treatment accordingly inhibited the key mediator levels of ER stress-induced apoptosis, which were consistent with that of 4-PBA. However, ER stress inducer Tunicamycin exhibited the opposite effects against 23BB and 4-PBA by immunoblot analysis (Figure 9B,D).

## Discussion

For several forms of AKI, therapeutics for kidney injury are not useful due to the unpredictable and intrinsic disease nature. On the other hand, cisplatin is a known chemotherapeutic agent with nephrotoxicity whose administration is scheduled and thus the development of renal protective strategies is feasible [23]. HDAC6 has been confirmed to contribute to cisplatin-induced AKI and selective inhibition of HDAC6 activity might be a potential strategy [24,25]. In our previous study, pretreatment of HDAC6i 23BB alleviated renal tubular damage against rhabdomyolysis-induced AKI. In the study, pharmacological inhibition of HDAC6 by 23BB treatment significantly attenuated acute kidney function and renal tubular damage in cisplatin-induced AKI. Our results indicated that 23BB inhibited HDAC6 activity to regulate tubular epithelial cell apoptosis via the inactivation of ER stress in the kidneys of cisplatin-induced AKI.

HDAC6 is a unique histone deacetylase harboring two active N-terminal deacetylating domains and a C-terminal ubiquitin-binding domain [26,27]. HDAC6, which is primarily expressed in the cytoplasm, clears the acetyl group from lysine residues in a number of non-histone substrates, including α-tubulin, stress granules and HSP90 [14,28,29]. Aberrant expression of HDAC6 is involved in several physiological processes such as stress response (ER stress), apoptosis and autophagy that play crucial roles in tumors, neurodegenerative disorders, inflammation and kidney diseases [15,30,31]. Compound 23BB as a highly selective and potent HDAC6 inhibitor with the IC_50_ of 17 nM has been synthesized and evaluated against anti-tumor activity in our laboratory [18]. In the study, treatment of HDAC6i 23BB at a dose of 40 mg/kg/d for 3 days effectively alleviated cisplatin-induced AKI and significantly suppressed tubular epithelial cell HDAC6 activity by the up-regulation of acetyl histone H3 in the injured kidney tissues.

Apoptosis is usually a response to cell microenvironment [32]. Apoptosis requires the lethal molecules activation and the inactivation of prosurvival ones [33]. Apoptotic pathways in the tubular epithelium could be induced by caspase cascade activation, mitochondrial injury, ER stress, etc. Apoptosis also promoted the loss of renal epithelial cells that characterized AKI [11,34,35]. Accumulating evidences demonstrated that the activation of caspase 3 was predominant which responsible for renal tubular cell apoptosis in cisplatin-induced AKI [13,36]. In our study, selective blockage of HDAC6 activity by 23BB notably reduced the number of TUNEL-positive cells, inhibited the cleavage of caspase-3 and up-regulated BCL-2, BCL-XL level in the cisplatin-injured kidneys and cisplatin-stimulated HK-2 cells. Therefore, HDAC6i 23BB could inhibit tubular epithelial cell apoptosis in the pathogenesis of cisplatin-induced AKI.

ER stress is a cellular stress response to the unfolded or misfolded proteins accumulation in the ER lumen, which is also called the UPR [6]. Multiple stimulatory signals or conditions could trigger ER stress in AKI, including hypoxia, mutant protein aggregation, energy deprivation and metabolic dysfunction [7]. Overwhelming ER stress induced apoptosis via three typical pathways: PERK, ATF6 and IRE1 [5,9]. Under UPR, PERK was released from its chaperone GRP78 to permit eIF2α phosphorylation, leading to the further activation of ATF4 and CHOP [37]. Active IRE1 targeted the downstream XBP1 and JNK that resulted in apoptosis [38,39]. In addition, caspase 12, which was only expressed in rodents, was another key molecule that responded to ER stress and induced the cleavage of caspase 3 to initiate apoptosis [38,40].

Considerable evidences indicated a link between HDACs and ER stress in lymphoma, breast cancer and Duchenne muscular dystrophy, etc [17,41,42]. Class I HDACs could localize to ER, bind to GRP78 and selectively activate UPR. HDAC6 in the cytoplasm also contributed to the acetylation of ER-localized chaperone GRP78 [17,19,24]. In our study, pharmacological inhibition of HDAC6 by 23BB markedly suppressed ER stress, as evidenced by the decreased protein expression of GRP78 and XBP1. The UPR was also inhibited by HDAC6i 23BB as indicated by the reduced expression of p-PERK/p-eIF2α/ATF4, ATF6 and IRE1 proteins. Moreover, HDAC6i treatment down-regulated the mediator expressions of ER-induced apoptosis, including CHOP, p-JNK and caspase 12. Taken together, the renoprotective effect of HDAC6i 23BB against tubular epithelial cell apoptosis was mediated via the inhibition of ER stress.

However, several limitations of the study were objective and need our further investigations. First, we did not explore the detailed relationship of 23BB-mediated HDAC6 activity and ER stress-related key proteins including PERK, ATF6 and IRE1. Second, siRNA-mediated silencing of HDAC6 and ER stress-related PERK, ATF6 and IRE1 should be performed for cisplatin-induced HK-2 cells to investigate the action of mechanism of 23BB. Finally, the location of 23BB-mediated HDAC6 protein in kidney tubules such as proximal tubular, distal tubular and collecting duct need to be precisely identified by specific markers.

The most significant thing was that we confirmed novel HDAC6i 23BB alleviated cisplatin-induced AKI and selective inhibition of HDAC6 was a promising strategy for the treatment of AKI. These findings also indicated that 23BB suppressed HDAC6 activity to reduce renal tubular cell apoptosis via the inhibition of ER stress in cisplatin-induced AKI.

## Supplementary Material

Supplementary Figures S1-S5 and Table S1

## Abbreviations

AKI: acute kidney injury
ATF6: activating transcription factor-6
BUN: blood urea nitrogen
ER: endoplasmic reticulum
HDAC6: histone deacetylases 6
HDAC6i: HDAC6 inhibitor
IRE1: inositol-requiring enzyme-1
JNK: c-Jun N-terminal kinase
PAS: periodic acid-Schiff
PERK: double-stranded RNA-activated protein kinase-like ER kinase
sCr: serum creatinine
XBP1: splicing of X-box binding protein 1
