# Supplementary material for: 2-Methylquinazoline derivative 23BB as a highly selective histone deacetylase 6 inhibitor alleviated cisplatin-induced acute kidney injury
Source: Biosci Rep. 2020 Jan 17;40(1):BSR20191538. doi: 10.1042/BSR20191538 (PMC6970081; doi:10.1042/BSR20191538)
Supplement: Supplementary Figures S1-S5 and Table S1 [file BSR-2019-1538_supp.pdf]

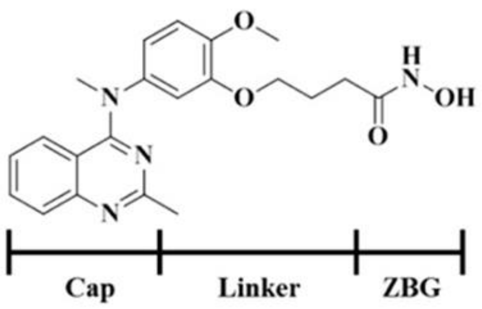

23bb

Oral bioavailability *F* = 47.0% (rats);

HDAC1 = 422 nM; **HDAC6** = 17 nM; **HDAC8** = 3398 nM.

Figure S1. Chemical structure and biological activity of 23BB.

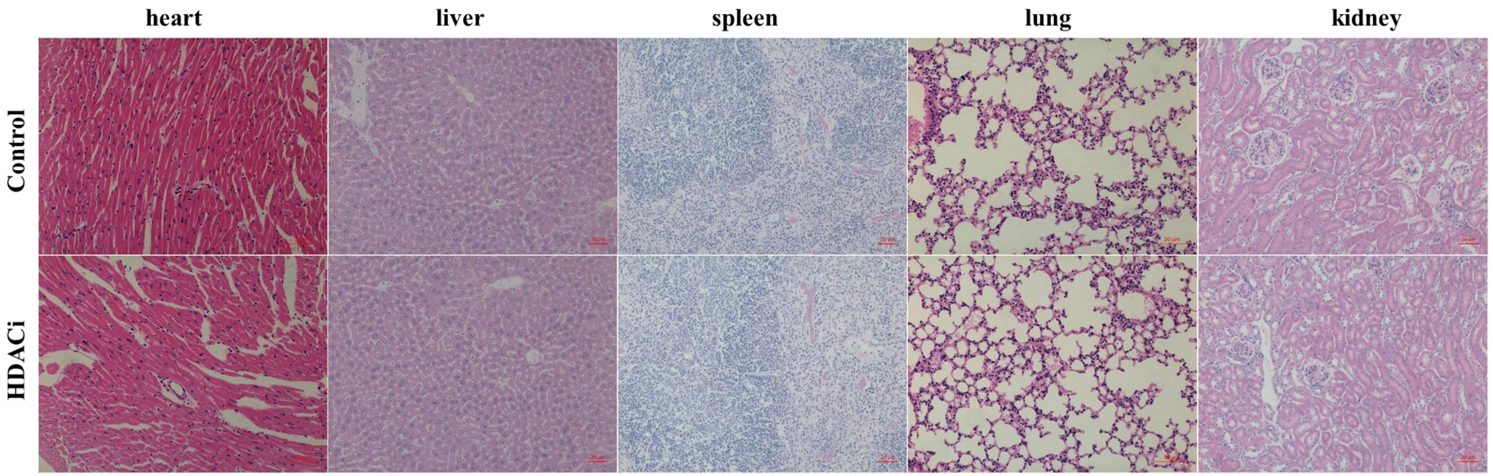

Figure S2. No pathologic changes of HDAC6i 23BB on Heart, Liver, Spleen, Lung and Kidney tissues. HDAC6i was orally administrated to C57 mice at a dose of 40 mg/kg/d for 3 days.

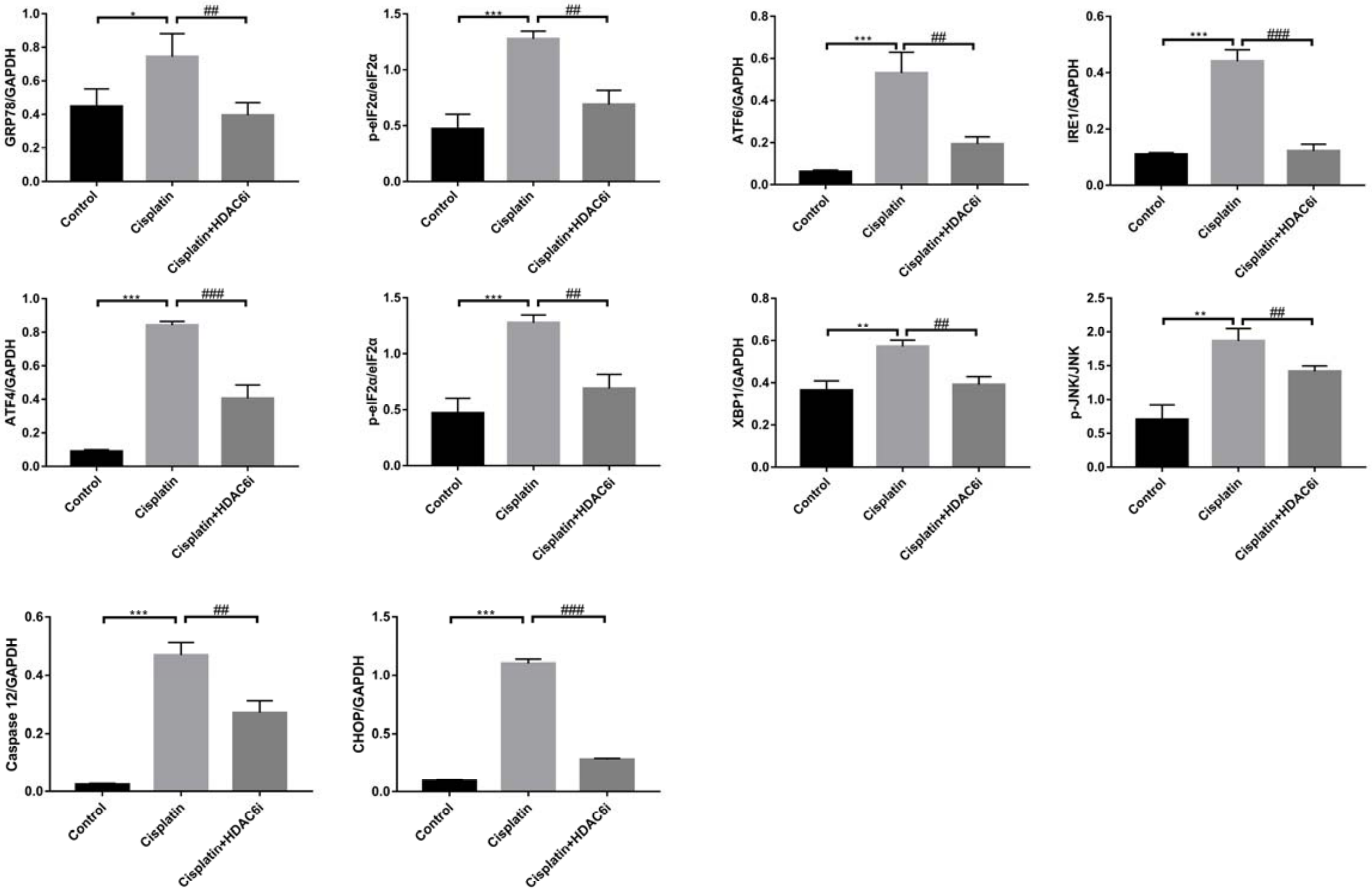

Figure S3. Kidney proteins analysis. The kidney tissue lysates were subjected to immunoblot analysis. Expressions of proteins were quantified by densitometry and normalized with GAPDH. Data are represented as the means±SE (n=3). *P<0.05, **P<0.01, ***P<0.001 *vs.* Control; $^{\#\#}$P<0.01, $^{\#\#\#}$P<0.001 *vs.* Cisplatin.

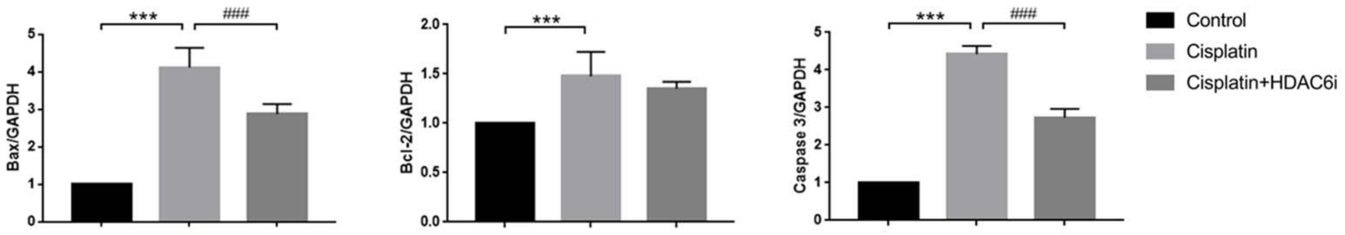

Figure S4. The mRNA expression of BAX, BCL-2 and Caspase 3 in the kidneys of cisplatin-induced AKI. All data are represented as the means±SE (n=3). ***P<0.001 vs. Control, ###P<0.001 vs. Cisplatin.

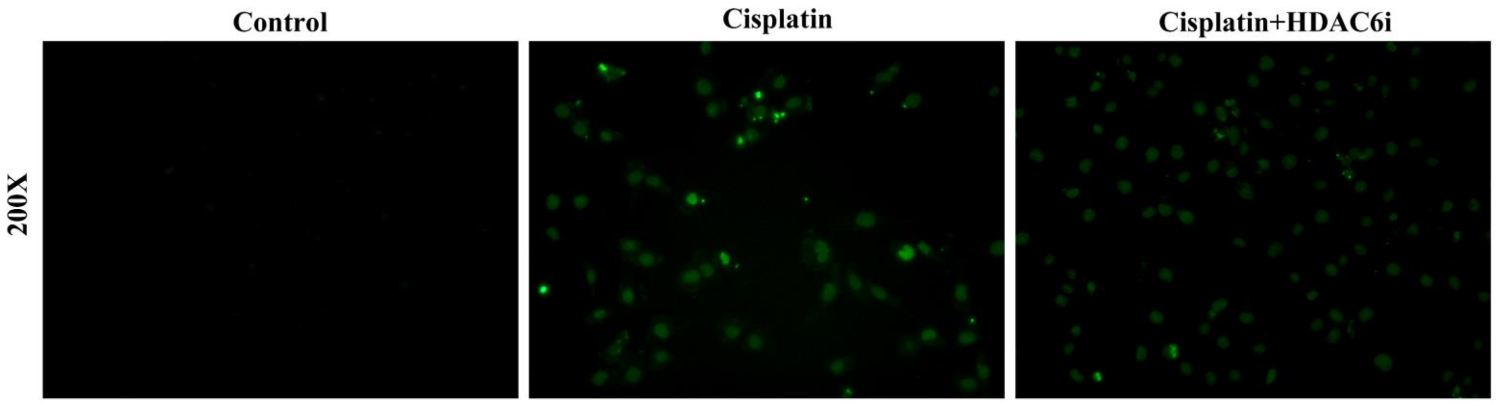

Figure S5. TUNEL staining of cisplatin-stimulated HK-2 cells. HK-2 cells were incubated with HDAC6i 23BB at 20 nM 30 min prior to cisplatin treatment (20 μg/ml) for 24 h.

Table S1. Primer sequences

Target	Forward	Reverse
GRP78	5′-ACACTTGGTATTGAAACTGTGG-3′	5′-GATCTGAGACTTCTTGGTGG-3′
IL-1β	5′-TGGGCCTCAAAGGAAAGAAT-3′	5′-CAGGCTTGTGCTCTGCTTGT-3′
IL-6	5′-ACAACCACGGCCTTCCCTACTT-3′	5′-CACGATTTCCCAGAGAACATGTG-3′
CHOP	5′-CTTCTCTGGCTTGGCTGACT-3′	5′-TCCCTTGGTCTTCCTCCTCT-3′
PERK	5′-GCCGACGATCAAATGGAAGC-3′	5′-ACCTGACTGTGATCTGCGTG-3′
BCL-2	5′-TGTGAGGACCCAATCTGGAAA-3′	5′-TTGCAATGAATCGGGAGTTG-3′
BAX	5′-GATCAGCTCGGGCACTTTAG-3′	5′-TTGCTGATGGCAACTTCAAC-3′
ATF6	5′-CCTTCGACCAGTCGGGTTTG-3′	5′-CTGTCCCGGAAAAGGCATCC-3′
ATF4	5′-CCTTCGACCAGTCGGGTTTG-3′	5′-CTGTCCCGGAAAAGGCATCC-3′
KIM1	5′-ACATATCGTGGAATCACAACGAC-3′	5′-ACTGCTCTTCTGATAGGTGACA-3′
NGAL	5′-GCAGGTGGTACGTTGTGGG-3′	5′-CTCTTGTAGCTCATAGATGGTGC-3′
GAPDH	5′-GTATGACTCCACTCACGGCAAA-3′	5′-GGTCTCGCTCCTGGAAGATG-3′
